# A feasibility randomised waitlist-controlled trial of a personalised multi-level language treatment for people with aphasia: The remote LUNA study

**DOI:** 10.1371/journal.pone.0304385

**Published:** 2024-06-14

**Authors:** Lucy Dipper, Niamh Devane, Rachel Barnard, Nicola Botting, Mary Boyle, Lin Cockayne, Deborah Hersh, Carla Magdalani, Jane Marshall, Kate Swinburn, Madeline Cruice

**Affiliations:** 1 Department of Language and Communication Science, School of Health and Psychological Sciences, City, University of London, London, United Kingdom; 2 Wolfson Institute of Population Health, Queen Mary University of London, London, United Kingdom; 3 Montclair State University, Montclair, New Jersey, United States of America; 4 Curtin School of Allied Health and EnAble Institute, Curtin University, Perth, Australia; Utah State University, UNITED STATES

## Abstract

**Background:**

Stroke survivors with aphasia want to improve their everyday talking (discourse). In current UK practice, 90% of speech and language therapists believe discourse assessment and treatment is part of their role but are hampered by barriers in resources, time and expertise. There is a clinical need for well-articulated discourse assessment and treatments. LUNA is a multi-level treatment targeting words, sentences and discourse macrostructure in personal stories that addresses this clinical need.

**Objectives:**

This study aimed to assess the feasibility and acceptability of LUNA trial procedures in a randomised waitlist-controlled trial; and to evaluate preliminary efficacy.

**Methods:**

This paper reports a phase II, waitlist-controlled, proof-of-concept feasibility trial. Participants with chronic aphasia (n = 28) were recruited from the community and randomised to an Immediate (n = 14) or Delayed (n = 14) group. LUNA treatment was delivered twice weekly for 10 weeks via the videoconferencing technology, Zoom. Feasibility was assessed in terms of participant recruitment and retention, adherence, missing data, and treatment fidelity. Preliminary treatment efficacy was assessed in terms of between group differences in outcome measures relating to discourse, language, and psychosocial state.

**Results:**

The remote LUNA trial was feasible: 85% of those eligible consented to the trial; trial retention was 86%; 87% of treatment sessions were delivered as scheduled, and 79% of participants completed 80%+ of the treatment programme; data was missing only for participants who withdrew; treatment fidelity was high at 92% adherence; and only one clinical outcome measure demonstrated ceiling effects. ANCOVA analysis of the clinical outcome measures revealed group differences with medium and large effect sizes, indicating, improvements in the production of words, sentences, discourse macrostructure, overall language functioning (WAB-R), and psychosocial state (VAMS) following LUNA treatment. For most outcomes measured, similar treatment benefits were suggested in a secondary, non-parametric analysis.

**Conclusions:**

Large-scale evaluation of the clinical efficacy and cost-effectiveness of LUNA is warranted and supported by these findings.

**Trial registration:**

Clinical trials registration: NCT05847023 (clinical trials.gov).

## Introduction

Stroke is a leading cause of long-term disability worldwide [1], and approximately a quarter of stroke survivors will experience chronic aphasia [2], a condition where communication is impacted with far-reaching consequences [3]. Aphasia affects the person’s abilities in speaking, listening, reading and writing, and has a negative impact on family and family roles, friendships, work, and access to healthcare and community life [4]. People with aphasia specifically want to improve their everyday talking—which is also referred to as ‘connected speech’ or ‘discourse’—in their rehabilitation with speech and language therapists [5]. Discourse is defined as a unit of language bigger than a sentence [6]; it is complex and requires processing multiple levels of language, including word retrieval, sentence construction, and adherence to an overarching discourse macrostructure. Discourse also has a key role in conversation [7]. For these reasons, discourse assessment has been identified as an ideal measure of functional communication in speech and language therapy (SLT) trials [8]; and improved discourse is a prioritised outcome for people living with aphasia [5].

The use of discourse assessment and treatment is gaining research interest and is now recommended in best practice guidelines [9]. However, conceptual and methodological issues remain [10]. There is a lack of consensus on how to define and assess discourse in the SLT field. SLTs surveyed across five countries defined discourse analysis differently [11]. International effort to establish a core outcome measure of functional communication for aphasia rehabilitation research did not initially reach a consensus [12], and more than 500 different measures of discourse have been identified in reviews [10, 13, 14]. Although the majority of SLTs believe discourse analysis is part of their professional role [15], there are practical barriers in assessing discourse that limit use in clinical practice. For example, a survey of UK SLT practice (n = 211) revealed that although 30% of SLTs collected discourse samples, only 5% of SLTs regularly transcribed them, and SLTs lacked relevant training and skills in interpreting discourse assessment findings [15]. Transcription is important because it allows for detailed analysis and subsequent relevant clinical management. It is especially important for personal narratives where the content of the discourse cannot be predicted. Despite its central role in everyday talk, the transcription, analysis and treatment of discourse is not widespread in UK NHS SLTs’ routine practices. There is a clinical need for well-articulated discourse assessments and treatments that are straightforward for clinicians to use.

While there is an evidence base for word and sentence treatments [16, 17], the evidence base for discourse treatments is only emerging with a recent systematic review [13] synthesizing 25 studies reporting on 127 participants and categorising discourse treatments into 5 different types. Although there was a wide range of different beneficial outcomes across these diverse treatments (including improved words, sentences and discourse macrostructure), the three studies showing most promise for improving multiple aspects of discourse reported a multi-level approach to treatment [18–20].

### LUNA treatment

This paper describes a novel discourse treatment for aphasia called, Language Underpins Narrative in Aphasia (LUNA). LUNA is a manualised, theory-based [21], codesigned [22] multi-level discourse intervention, which aims to facilitate the telling of personal stories through word, utterance (sentence) and discourse macrostructure level activities. It integrates familiar treatments—semantic feature analysis, mapping therapy, story grammar—to provide flexible metalinguistic tools for improving people’s confidence and ability to express themselves through narrative. These treatment activities are integrated through the use of a personally chosen story on which to work during treatment. LUNA is distinct from previous multi-level treatments in its form of personalisation (the focus on a story that the person has selected and wants to tell to family and friends); its explicit focus on meta-linguistic awareness (activities are aimed understanding the person’s own language profile); and meta-cognitive awareness to support self-management.

LUNA is personalised in two ways. Firstly, there is personalisation in the subject material. Participants choose stories from their own lives that they want to share with others. Secondly, there is personalisation in the linguistic content. The participant chooses the words, sentences, and macrostructure they use to tell their story in collaboration with the therapist during treatment. There is evidence that therapy outcomes are enhanced when personalised content is included [23], and that this stimulates neural re-organisation [24]. In addition, the treatment of personal stories can have broader effects. The sharing of stories may help people to express themselves, and to interact and share more with family and friends [25–27]. LUNA is a meta-cognitive [28] and meta-linguistic [29] therapy, encouraging participants to reflect on their own thinking and language; to learn about the nature of language, and the detail of their own linguistic skills and impairments; and to practise using the new skills in everyday contexts. Ultimately, this means the use of personal stories may serve to increase motivation to engage with and complete the treatment programme—of relevance to discussions of feasibility and adherence described later in the paper.

LUNA was initially devised for face-to-face delivery. However, this study coincided with the 2020 COVID-19 pandemic. All assessment and treatment procedures were therefore adapted for remote delivery using videoconferencing technology, specifically Zoom. Research has demonstrated that people with aphasia can comply with remote assessment and treatment and find such procedures acceptable [30] and that remote treatment can have positive outcomes [31–33]. Remote delivery of multi-level discourse treatment for aphasia has not previously been trialled.

This proof-of-concept study comprised a phase II randomised controlled trial, comparing remote LUNA treatment with a waitlist control. It aimed to test the feasibility of trial procedures and explore indicative outcomes from LUNA treatment. Specifically, this study aimed to:

1. Test the feasibility of a definitive trial comparing remote LUNA with a waitlist control, using the following feasibility endpoints: a) participant recruitment and retention rates; b) adherence to treatment sessions; c) counts of missing data; and d) fidelity scores for treatment delivery.
2. Explore the appropriateness of the trial outcome measures, as indicated by the level of variability of scores, missing data, and floor and ceiling effects.
3. Investigate preliminary efficacy by comparing outcomes on discourse, language, and measures of psychosocial state across participants who have and have not received the LUNA intervention.

## Materials and methods

### Trial design

The study was a single-blind, waitlist, randomised, controlled, phase II, proof-of-concept, feasibility and acceptability trial of remote LUNA for people with chronic post-stroke aphasia.

This study was granted ethical approval by the City, University of London, School of Health Sciences Research Ethics Committee (ETH1920-0210) in February 2020; similarly, approval was granted in June 2020 for amendments (ETH1920-1651) following the COVID-19 national lockdowns, prior to the trial starting recruitment. The trial sponsor was City, University of London.

### Participants

Twenty-eight participants were recruited to the remote LUNA study between 16/06/2020 and 06/08/2020. Twenty-eight was an intentional over-recruitment on a target of at least 24 participants, to mitigate for possible attrition. The intention was to ensure a sample size of 24 (12 treated, 12 control) following recommendations for feasibility trial sample sizes [34, 35]. Inclusion criteria were adults (18+ years); diagnosis of ischaemic or haemorrhagic stroke; and aphasia due to a stroke that occurred at least 12 months prior to recruitment. Additionally, participants were literate and fluent users of English prior to their stroke (self-reported), with adequate hearing and vision with aids or glasses (for example to see pictorial and written assessment and treatment materials). Participants were required to have access to a computer or tablet and an internet connection. They needed to be able to download and access Zoom, either independently or with the support of a friend/neighbour/family member.

Participants were excluded if they were receiving speech and language therapy elsewhere or participating in any other aphasia treatment research project for the duration of the study. Usual stroke supports, such as voluntary sector support groups, could proceed. Although many of these support services were curtailed due to COVID-19, some moved online. Participants with severe aphasia, as defined as a score of 7 or less on the Frenchay Aphasia Screening Test (FAST) [36], were excluded. This criterion was applied because remote LUNA was designed for people with some verbal output. It was also judged that people with severe aphasia would struggle to manage remote delivery. Participants were also excluded if they had a secondary cognitive diagnosis such as dementia. This was established via self-report and/or the confirmation of the referring group co-ordinator and/or by expert clinical judgment of research project staff. Screening and recruitment were completed by experienced SLT members of the research team (authors KS and MC).

Participants were a volunteer sample recruited by advertising the study through UK-based stroke support groups, signposting people to the dedicated project website or self-referral. Self-referrals were accepted from anywhere in the United Kingdom. All recruitment, assessment, treatment, and interview sessions were conducted online using Zoom. All participants gave written consent. All participant information sheets and consent forms were made accessible to people with aphasia following evidence-based recommendations [37]. Recruitment began on 16 June 2020 (first screening) and data collection finished on 28 April 2021 (final assessment).

### Intervention

The LUNA treatment is specified in the TIDIER checklist (see S1 Checklist). Before treatment started, participants were supported to choose two personal narrative monologues to share. They were given about a week to consider their choice and then both narratives were elicited at the beginning of the first assessment session, under controlled conditions, following a set procedure. Participants then decided which of the two narratives they wished to work on in treatment sessions. This choice was shared with therapists, and the chosen narrative was transcribed, analysed and deconstructed to identify potential treatment targets, ahead of the first treatment session.

Remote LUNA comprised 20 hours of treatment, 2 sessions per week of 60 minutes each, for 10 weeks. A set-up week preceded treatment, where the SLT and participant met for an hour to agree on goals–the deconstructed narrative was used as a basis for this discussion. This resulted in an intervention lasting 11 weeks, consisting of 21 hours of treatment in total. In week 2–11, the chosen personal narrative was progressively re-built through integrated word, phrase, clause, multi-clause, and discourse macrostructure treatment activities. Treatment targeted three language levels: word (wks 1–4); utterance (weeks 5–7); and macrostructure (weeks 8–10).

All sessions were delivered over Zoom. One session per week was delivered by a qualified SLT and the other session was delivered by an assistant: a student SLT (SSLT). Both the SLT and SSLT followed the treatment manual and received guidance via remote supervision. Linked ‘challenge tasks’ promoted generalisation outside of treatment sessions.

A team of three experienced SLTs and twelve SSLTs delivered remote LUNA to the 28 participants. Alongside guidance from the treatment manual, SLTs received six days of remote training across a three-week period prior to implementing treatment, in addition to weekly remote group supervision from a clinical linguist (author LD) throughout the trial. SSLTs received fourteen hours of remote training and received a mixture of 1:1, paired and group supervisions remotely throughout the trial. Each participant worked with the same SLT and SSLT for treatment for the duration of the study (there were different screening and Assessor SLTs for recruitment and assessment–see below).

### Feasibility outcomes

Feasibility of remote LUNA was tested in terms of participant recruitment and retention, adherence throughout the study, and missing data. To inform a future trial, treatment fidelity, appropriateness of outcome measures and estimated sample size were also explored. Six feasibility endpoints were outlined:

- Feasibility of recruitment and retention to the trial: Data comprised of counts/ proportion of those who expressed interest, were screened and deemed eligible, those who consented, attrition and reasons for attrition if known.
- Adherence: data comprised of number/proportion of treatment sessions attended as scheduled, and percentage completion of the LUNA treatment programme; reasons for non-attendance.
- Missing Data: Data comprised of attrition rates and counts of other missing data.
- Assessment of treatment fidelity through ratings of provider adherence to the LUNA manual/essential elements; reliability of the rating procedure was checked and whether scores were affected by the treatment provider, treatment level or group allocation.
- Appropriateness of outcome measures: indicated by the level of variability of scores, missing data, and floor and ceiling effects.
- Estimate of sample size for a future trial: based on a preliminary power calculation using WAB-R effect sizes.

### Treatment fidelity

Remote LUNA yielded a total of 560 hours of Zoom-recorded treatment sessions (28 participants x 20 sessions), and a sample of 10% of sessions (56hrs) was selected for review. Sample selection was stratified (by authors LD and MC)–it was organised to ensure that a range of providers and treatment levels were sampled but was otherwise random (i.e. done without reference to session content or participant details). Treatment fidelity (TF) was assessed by evaluating providers’ adherence to the treatment manual (as determined by SLT student raters) using a TF checklist of essential elements of LUNA.

The checklist was developed iteratively with the research team, co-designers with aphasia, co-designer SLTs, LUNA therapists, and research students. The final checklist (Table 1) comprised 12 items. These were used by all treatment providers, during the treatment phase, as a self-reflective tool after completing sessions. The same checklist was then used post-treatment phase by SLT student raters to evaluate providers’ adherence to the treatment manual. Two of these students were not part of the team that delivered intervention, and two were. The latter two students did not evaluate their own sessions and so all four students were considered unbiased raters.

**Table 1:** LUNA treatment fidelity checklist items.

Item	Description*
1	The SLT/SSLT promotes partnership and collaboration
2	Clear goals orientation in the session
3	Client is actively involved in making decisions in the session
4	Emphasis on the client’s understanding (meta-awareness)
5	Evidence of personalisation
6	Good therapeutic practice
7	Session relates to 1 of the 3 LUNA levels (word, sentence, discourse)
8	Both story and non-story treatment targets are used in the session
9	Flexibility and/or responsiveness is evident in the session
10	Evidence of supportive performance monitoring i.e., feedback and reflecting on progress
11	Work in the session is explicitly linked to the challenge tasks
12	Evidence that the manual is being followed

*Definitions and examples of each item that appear in the full checklist have been omitted here for brevity.

In the post-treatment phase, SLT student raters evaluated fidelity by marking each of the 12 items as either present or absent [38] including additional qualitative notes to justify their decisions. Fifty-six hours (10%) of treatment sessions were viewed by four research students. These raters received training (4 hours) which comprised group and independent viewing and discussion, and independent benchmarking. Training was carried out on six representative sessions, selected to include: word, sentence, discourse macrostructure treatment activities; and delivery by SLTs and SSLTs. Percent agreement on benchmarked sessions was 72% (26/36 items) with most discrepancies on items 8, 10 and 11. These were discussed, with refinements added to the checklist.

Fourteen sessions were allocated to each of the four raters (total 56 sessions) and assessed independently. Eight (8) of 56 sessions were subjected to intra-rater reliability checks with ratings separated by a period of at least 1 month, and a further eight (8) of 56 sessions subjected to inter-rater reliability checks. Reliability was determined by calculating percentage agreement with agreement interpreted as high if >70% [39].

### Clinical outcome measures

Participants completed assessments at three time points: T1 (weeks 1 & 2), T2 (weeks 13 & 14) and T3 (weeks 25 & 26). Only efficacy outcomes at T2 are reported here, to enable a comparison of treated (i.e. Immediate treatment group) and untreated (i.e Delayed treatment group) participants. Participants in the Immediate group received LUNA treatment between T1 and T2. Participants in the Delayed group received treatment between T2 and T3, but their efficacy results are not reported in this paper. Participants were recruited to the study in two waves to allow for appropriate staffing.

Feasibility findings from all three timepoints are presented for completeness. For the preliminary efficacy evaluation, we report clinical outcomes from T1 and T2 only, comparing the experimental (Immediate) group who had received treatment at this point to the control (Delayed) group who had not yet received treatment.

At each timepoint, assessment was completed by LUNA Assessors (n = 2) who were qualified SLTs who were kept blinded to participant treatment group allocation throughout the study. Assessment processes were adapted for online delivery and manualised. Assessors undertook this development work across a 6-week period prior to assessing participants, also using this time to undertake training and practice remote assessment with 3 people with aphasia who were part of the LUNA PPI Advisory group. For the discourse analysis, Assessors were given weekly training over a two-month period (including training with a clinical linguist and self-directed exercises). In addition, they received regular supervision from an SLT (author MC) during the assessment phases and from a clinical linguist (author LD) during the narrative assessment phase.

### Personal narratives measure (LUNA discourse protocol)

Participants produced two personal narratives at each assessment point, which were recorded, transcribed, and analysed by according to the LUNA Research Discourse Analysis Protocol. Several discourse metrics were calculated from the analysis (see S1 Appendix), with the selection made during codesign session with the SLTs and guided by: use of a measure in the systematic review [13], the psychometric properties [40, 41] of the measures, and the appropriateness of the measure for measuring change after LUNA treatment. A novel measure ‘narrative words’ was designed by the research team which, while similar to Correct Information Units (CIUs), was intended to be more clinically feasible as an analysis. Number of narrative words was proposed as the primary clinical outcome measures. Other discourse metrics included: number of CIUs, percentage of CIUs, and number of CIUs/minute (following the protocol of Nicholas and Brookshire, 1993); number of narrative words, percentage of narrative words, and number of narrative words/minute; number of complete utterances and percentage of complete utterances; number of multiclause utterances and percentage of multi-clause utterances; predicate argument structure (PAS) score; a Story Grammar score; and a count of the number of clear reference chains (see S1 Appendix).

### The Western Aphasia Battery-Revised (WAB-R) [42]

The WAB-R is a performance-based outcome measure assessing speaking, auditory comprehension, naming, and repetition across four sections. It classifies aphasia type and generates an aphasia severity score between 0–100, the Aphasia Quotient (AQ), where a score of 0–25 is very severe, 26–50 is severe, 51–75 is moderate, 76+ is mild. A cut-off score 93.8 and above is considered "normal or nonaphasic" (pg. 91, [42]). The AQ score was used in the analysis. It was standardised on people with aphasia (n = 150) and controls (n = 59) [43]. Internal consistency and interrater reliability are good [44]. It is internationally used as part of the core outcome set for aphasia trials [12] and has been validated for remote online delivery [45].

### The Communicative Participation Item Bank (CPIB)–General short form [46]

The CPIB is a 10-item patient-reported outcome measure (PROM). Patients rate the level of interference caused by their condition for each item, on a 4-point scale. Items ask, for example, how much the condition interferes with communicating with people known to the person with aphasia, with people not known to them, when giving someone detailed information, and when communicating as part of a small group. Scores are converted to a summary score which ranges between 0–30 where a high score is favourable, representing little interference from the health condition. The summary score was used in the analysis. The measure was designed for community-dwelling adults with spasmodic dysphonia but was adapted for aphasia with a representative sample. The short form is appropriate and valid for people with aphasia [47].

### The Communication Confidence Rating Scale for Aphasia (CCRSA) [48, 49]

The CCRSA is a 10-item PROM. Patients rate their confidence in communicating in different contexts on a scale of 0–100. Scores are converted to derive a total score of between 10–40, where 40 represents feeling very confident in communicating. The total was used in the analysis. It is the only communication confidence measure in the field and is increasingly used in treatment studies. It was validated on 47 people with aphasia from different treatment settings. The psychometric properties of sensitivity to change and reliability (inter- and intra-) remain to be established [49].

### The Assessment for Living with Aphasia (ALA) [50]

The ALA is a 45-item pictographic PROM assessing aphasia-related quality of life and was developed by an internationally leading aphasia charity in Canada. Questions cover four domains relating to living with aphasia (language impairment, participation, personal factors, and environmental factors) and there is a summary question relating to the overall impact of aphasia. The total scores of these 5 items are divided by 37 to create a single mean score. The mean score ranges between 0–4, where 4 represents a perception of good quality of life. The mean score was used in the analysis. Acceptable construct validity and reliability have been established [51].

### Visual Analogue Mood Scales (VAMS)–Sad [52]

Following feedback from LUNA advisors with aphasia (and supported by the research team), a single item mood measure, the Visual Analogue Mood Scales (VAMS) Sad scale was added to all testing time points. Scores range between 0–100, with 100 representing a maximal level of sadness and zero representing a minimal level (or absence) of that mood. It has been used successfully in aphasia studies [53, 54] and takes three minutes to complete. It is accessible and appropriate to be used with stroke survivors who have aphasia [55]. Content validity [52, 56] and test re-test reliability [57] have been established.

### Randomisation

Stratified random sampling was used. After T1, participants were classified into two groups: group (i) ‘mild’ and group (ii) ‘moderate’ aphasia severity based on WAB AQ score. Randomisation was carried out by a research team member (author NB) who was blinded to severity by use of the group labels (i) and (ii), and who was also blinded to screening and assessment results and had no knowledge of the participants. Participants were randomised to the immediate or delayed condition by the following method: for each group (i/ii, i.e. mild/moderate), participant numbers were written on identical pieces of paper which were then folded in half; these were placed in a box and shaken, then pulled out in a random order; in alternating fashion, each number was allocated to the Immediate group or the Delayed group.

### Blinding

Limited members of the research team were aware of participant treatment group allocation (Immediate/Delayed). These members were the joint principal investigators, project manager, treating SLTs and SSLTs, and the qualitative researcher. Other members of the research team (n = 6) were kept blinded to group allocation. This included, most importantly, the Assessors (n = 2) who were qualified SLTs kept blinded to group allocation throughout the study–this included them agreeing to delete their social media accounts for the duration of the trial in order to remove the risk of unblinding in that context.

Recruitment sessions were organised by the project manager and one principal investigator, and assessors had no access to participant files or details that would reveal group allocation. Remote working, imposed by COVID-19, also ensured that assessors had minimal contact with the unblinded members of the research team, beyond formal supervision with the Principal Investigators. Participants were instructed not to reveal their group allocation to assessors during assessment sessions. A log was kept of any instances of unblinding and near misses, with the reason for the unblinding.

### Analyses

Regarding feasibility, analyses were descriptive to ascertain feasibility endpoints such as recruitment and attrition. Adherence, in terms of sessions delivered as scheduled and participants’ completion of the treatment programme, was recorded as a percentage of sessions. With respect to treatment fidelity, a score was calculated for each item as a percentage of items marked as present and interpreted as high if 80–100% and low if 50% or lower (scores 51%-79% being medium) [58]. Fidelity findings were also examined in relation to treatment provider (SLT/SSLT), treatment level (word, sentence, discourse) and group (immediate, delayed).

Regarding clinical outcomes, a between-group comparison analysis was carried out. ANCOVA compared assessment scores of both groups at T2 (when the Immediate group had received treatment, but the Delayed group had not) in measures of discourse, language, and psychosocial state, using T1 scores as a covariate. These analyses were exploratory, examining whether the treatment showed promise of efficacy. An indication of treatment promise would be seen in a significant group effect favouring the immediate condition and/or effect size (partial eta squared: η2 ~ 0.01 = small effect; η2 ~ 0.06 = medium effect; η2 ~ 0.14 = large effect). Preliminary power calculations were conducted based on the effect sizes of the standardised language measure (WAB-R) to determine sample size for a future clinical efficacy trial of LUNA.

## Results and discussion

### Participants

**Fig 1:**
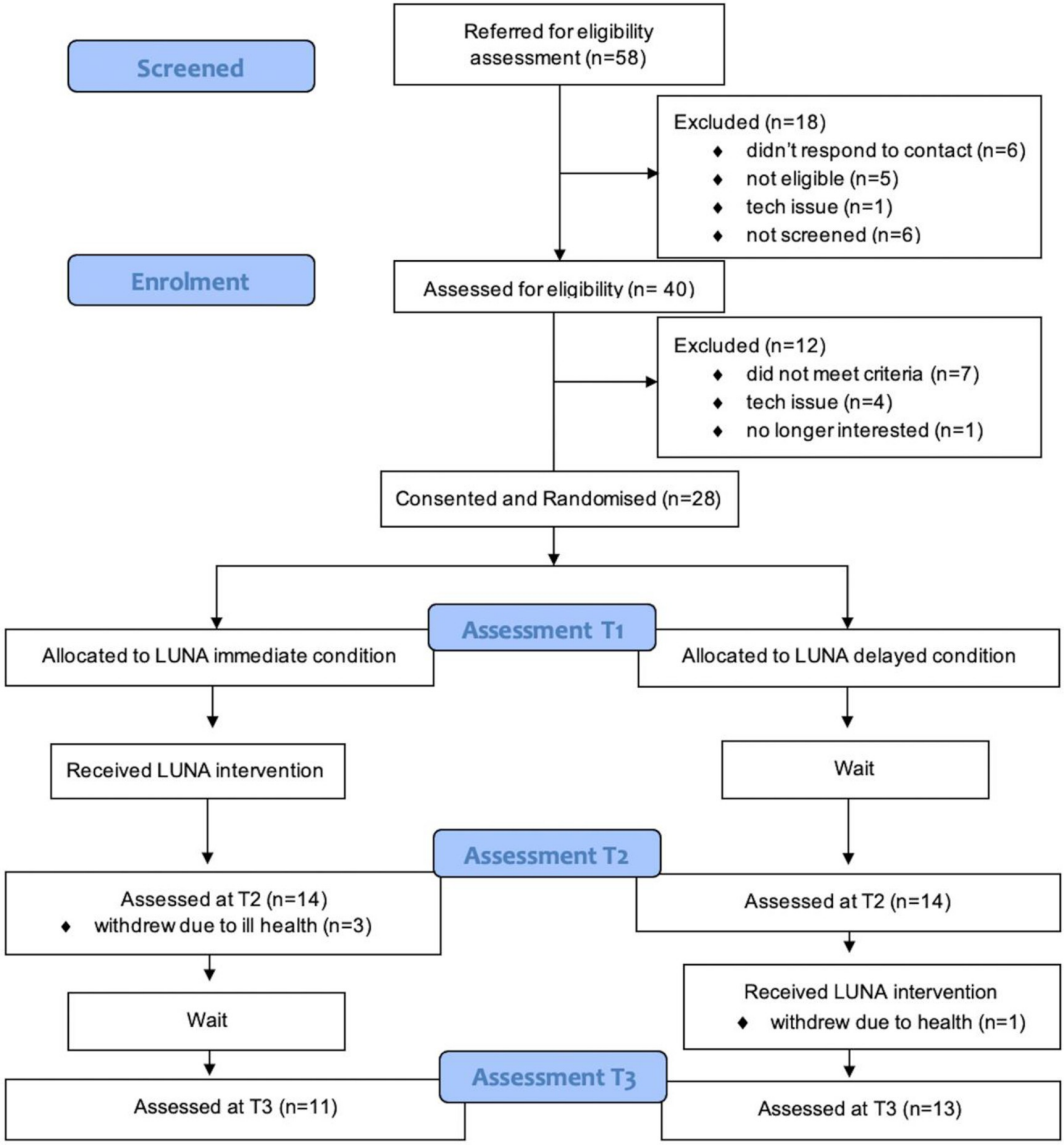
Participant flow diagram.

Twenty-eight (28) people with aphasia were recruited to the trial in a two-month period between 16 June 2020 and 6 August 2020. Fifty-eight (58) people expressed an interest, 40 people were screened using the FAST, and 28 were randomised (Fig 1).

Participants were on average ~60 years old, ranging from 34–83 years (See Table 2). They were predominantly from a White British ethnic group, university educated, and had held highly skilled positions in their working lives as measured by the Standard Occupational Classification [59]. All participants had English as their primary language with more than half the sample using more than one language but only three participants described an advanced ability in other languages. Participants came from two of the four UK countries and from seven of the nine regions in England, representing a large geographical spread. There were no participants from Wales and Northern Ireland, or from the West Midlands or the North East of England. See Table 2 for participant characteristics. Participants were on average 55 months post-stroke (range 14–181 months) and largely balanced between mild and moderate aphasia severity.

**Table 2:** Participant characteristics at baseline (T1).

	Immediate (n = 14)	Delayed (n = 14)	Total (n = 28)
**Age**	57.72 years	58.07 years	59.82 years
(range)	(41–83)	(34–82)	(34–83)
**Ethnicity**			
White British	13 (93%)	13 (93%)	26 (93%)
White other	1 (7%)	1 (7%)	2 (7%)
**Language**			
Mono-lingual	14 (100%)	11 (79%)	25 (89%)
Multilingual	0	3 (21%)	3 (11%)
**Education**			
Secondary	5 (36%)	5 (36%)	10 (36%)
Further	2 (14%)	0	2 (7%)
Higher	7 (50%)	9 (64%)	16 (57%)
**Occupation**			
1.Manager/Director	2 (14%)	6 (43%)	8 (29%)
2.Professional	3 (21%)	2 (14%)	5 (18%)
3.Associate Professional	3 (21%)	1 (7%)	4 (14%)
4.Administrative and secretarial	3 (21%)	3 (21%)	6 (21%)
5.Skilled trade	1 (7%)	0	1 (4%)
6.Caring and leisure	0	0	0
7.Sales and customer service	0	0	0
8.Machine operatives	1 (7%)	1 (7%)	2 (7%)
9.Elementary	0	0	0
(Retired)	1 (7%)	1 (7%)	2 (7%)
**Geographical Region**			
South East	5 (36%)	2 (14%)	7 (25%)
South West	5 (36%)	2 (14%)	7 (25%)
London	2 (14%)	3 (21%)	5 (18%)
East of England	0	3 (21%)	3 (11%)
Scotland	2 (14%)	1 (7%)	3 (11%)
East Midlands	0	1 (7%)	1 (4%)
North West	0	1 (7%)	1 (4%)
Yorkshire and the Humber	0	1 (7%)	1 (4%)
**Living status**			
Alone	4 (29%)	3 (21%)	7 (25%)
With partner and family	8 (57%)	9 (64%)	17 (61%)
With other family	2 (14%)	2 (14%)	4 (14%)
**Stroke/handedness information**			
Right handedness	8 (57%)	14 (100%)	22 (79%)
Left handedness	5 (36%)	0	5 (18%)
Ambidextrous	1 (7%)	0	1 (4%)
Right hemiplegia	9 (64%)	12 (86%)	21 (75%)
Left hemiplegia	1 (7%)	0	1 (4%)
No hemiplegia	4 (29%)	2 (14%)	6 (21%)
**Mean time post stroke in months**	59	55	55
(range)	(14–181)	(20–105)	(14–181)
**History of stroke**			
Single stroke event	12 (86%)	12 (86%)	24 (86%)
History of 2 strokes	1 (7%)	2 (14%)	3 (11%)
History of >2 strokes	1 (7%)	0	1 (4%)
**Aphasia severity**			
Not aphasic by WAB-R score**	2 (14%)	0	2 (7%)
Mild (76–94 WAB AQ)	6 (43%)	6 (43%)	12 (43%)
Moderate (51–75 WAB AQ)	6 (43%)	7 (50%)	13 (46%)
Severe (26–50 WAB AQ)	0	1 (7%)*	1 (4%)
**Aphasia Classification†**			
Broca’s	2 (14%)	2 (14%)	4 (14%)
Wernicke’s	2 (14%)	1 (7%)	3 (11%)
Conduction	5 (36%)	6 (43%)	11 (39%)
Anomic	3 (21%)	5 (36%)	8 (29%)
Not aphasic by WAB score**	2 (14%)	0	2 (7%)

Multilingual = participants who describe advanced or native ability in another language. WAB-R AQ = Western Aphasia Battery-Revised Edition, Aphasia Quotient score.

*The FAST was used at screening to screen out participants with severe aphasia, but at baseline testing one participant was classified within the severe range on the WAB-R.

** Although an AQ of 93.8 or above is suggested as a cut-off for aphasia diagnosis, we included participants who scored in the range 93–100 on the WAB-R because recent studies have shown people with such scores perform significantly differently to controls in discourse tasks. † Aphasia classifications not represented in this sample: global; isolation; transcortical motor; transcortical sensory

### Feasibility outcomes

#### a) Participant recruitment and retention

The remote LUNA study recruited 28 participants, which was 100% (28/28) of the target sample size. In brief, 48% (28/58) of those who expressed an interest, and 85% (28/33) of those who were eligible consented to participate in the trial (Table 3). See Fig 1 for further detail about reasons for exclusion at each stage. Four participants withdrew from the study due to ill health: three from the Immediate group following T2 testing, and one from the Delayed group after treatment but before T3 testing. Therefore, retention was 86% (24/28).

**Table 3:** Participant recruitment and retention.

	Proportion or Rate	Number
Proportion eligible of those identified	48%	28/58
Proportion eligible of those screened	83%	33/40
Proportion consented of those eligible	85%	28/33
Rate of eligible/month	14/month	28 (recruited in total in 2 months)
Proportion of withdrawals		
Overall:	14%	4/28
By group:		
• Immediate	11%	3/28
• Delayed	4%	1/28

#### b) Adherence

Eighty-eight percent (88%) of *assessment* sessions were attended as scheduled, i.e., at the time and date arranged, and for the scheduled length of time. The remaining 12% either needed an additional session in order to complete the intended assessment or needed a session to be rescheduled. Reasons for 12% not going ahead as planned included technical issues (in the majority of cases) and health or personal reasons.

Participants attended 87% of remote *treatment* sessions as scheduled. Reasons for 13% sessions not going ahead as planned were: the session was split across more than one session on the same day due to technical difficulties (31%); the session started late due to technological (29%) or other reasons (13%); ill health (10%); or was rearranged for a different day (17%). In terms of completion of the LUNA treatment programme, 54% participants (n = 15/28) attended 90–100% of the programme, 25% of the participants (n = 7) attended 80–89% of the programme, and 21% of participants (n = 6) attended 67–79% of the programme.

There were minimal differences between the Immediate and Delayed groups in terms of adherence, indicating that having to wait for treatment was not a significant factor.

#### c) Missing data

All (28/28) participants completed assessment sessions at T1 (baseline) and T2 (post-treatment for the Immediate group); and 86% (24/28) completed assessment sessions at T3, with four participants withdrawing due to ill health prior to T3. Completeness of data was also monitored at the item level, and data was either all present for assessments, or all missing (i.e. for those four participants at T3).

#### d) Treatment fidelity

The remote LUNA treatment was delivered as intended, with high adherence to the manual. 92% of items were marked as present (616/672). Half the checklist items had 100% adherence (items 1, 4, 5, 6, 7, 9), five items had >80% (items 2, 3, 10, 11, 12) and only one item on the checklist, item 8, had low adherence at 32%. This data is underpinned by 100% intra-rater reliability findings, and 98% inter-rater reliability findings. Treatment adherence was explored in more detail in relation to provider, treatment level, and group. Only 8% (56/672) of items were marked as absent. SSLT sessions had more items rated absent (63%, 35/56) than SLT sessions (38%, 21/56). Discourse level sessions had more items rated absent (43%, 24/56) than word (29%, 16/56) or sentence (29%, 16/56) sessions. There were more items rated as absent in the Immediate group (55%, 31/56) compared to the Delayed group (45%, 25/56).

#### e) Appropriateness of trial outcome measures

The outcome measures data was appropriate and usable. There was a change in mean scores over time in the expected direction, suggesting sensitivity to the effects of the treatment. No floor or ceiling effects were observed except for the VAMS-Sad where, at T1, T2, and T3, 17.9%, 42.9%, and 17.9% of participants scored the highest score possible (0; reflecting absence of sadness). There was no missing data due to participants not being able to complete measures, only from participant withdrawals.

Regarding unblinding, assessors were inadvertently unblinded for seven (7) of the 28 participants. For example, on one occasion a participant screenshared their calendar with an Assessor to find an assessment, inadvertently making treatment sessions appointments visible. On another occasion, the Assessor rather than SLT, SSLT or Project Manager was called for technological support when someone couldn’t access zoom for the treatment session.

### Clinical outcomes

Clinical outcomes were measures of discourse from personal narratives. Descriptive statistics are presented for the discourse measures in Table 4 and for the measures of language and psychological state in Table 5. At T1 (pre-treatment) there were no significant differences between groups (all p values > 0.3).

**Table 4:** Means and standard deviations for discourse measures.

	T1		T2	
	mean (SD)		mean (SD)	
	Immediate	Delayed	Immediate	Delayed
Narrative words:				
number	*428*.*1 (403*.*1)*	*450*.*8 (490*.*3)*	599.3 (388.1)	494.5 (543.8)
percentage	66.5 (10.2)	65.3 (14.4)	69.5 (13.5)	66.1 (14.2)
number per minute	41.96 (21.36)	49.52 (29.27)	45.44 (21.54)	58.27 (30.03)
Correct Information Units:				
number	372.4 (369.1)	*399*.*4 (440*.*0)*	532.0 (365.9)	435.8 (489.5)
percentage	61.1 (11.2)	62.1 (13.9)	64.2 (13.5)	62.7 (13.3)
number per minute	35.87 (20.06)	43.60 (27.09)	40.03 (20.68)	50.49 (27.33)
Utterances:				
number complete	*38*.*9 (37*.*1)*	*35*.*0 (40*.*0)*	54.6 (38.7)	38.6 (45.0)
% complete	59.0 (21.8)	54.1 (26.7)	66.9 (22.7)	51.1 (28.9)
number multiclause	*13*.*9 (15*.*8)*	*18*.*0 (23*.*0)*	22.1 (18.7)	21.1 (27.6)
% multiclause	20.7 (15.5)	25.2 (16.6)	27.6 (18.6)	24.6 (21.4)
Predicate Argument Structure	*1*.*8 (0*.*2)*	1.7 (0.3)	*1*.*8 (0*.*2)*	1.7 (0.2)
Story Grammar:				
number of elements	3.6 (1.6)	4.0 (2.0)	4.1 (1.2)	4.0 (2.0)
Clear reference chains:				
number of chains	8.9 (9.9)	7.1 (7.5)	13.7 (11.7)	7.9 (9.6)

nb: italics indicate *skewed data*.

**Table 5:** Means and standard deviations for measures of language and psychological state.

Scale [score range]	T1		T2	
	mean (SD)		mean (SD)	
	Immediate	Delayed	Immediate	Delayed
WAB-R AQ	76.44 (13.56)	73.20 (13.54)	77.86 (12.01)	72.47 (13.39)
[0–100]				
CPIB	13.14 (3.92)	10.07 (4.10)	13.71 (4.01)	12.00 (5.46)
[0–30]				
CCRSA	28.79 (5.54)	27.14 (4.22)	29.64 (3.95)	27.50 (4.47)
[10–40]				
ALA	2.66 (.54)	2.42 (.46)	2.79 (.55)	2.44 (.50)
[0–4]				
VAMS-Sad	12.98 (17.31)	13.46 (8.39)	6.88 (11.60)	15.39 (6.88)
[0–100]				

WAB-R AQ = Western Aphasia Battery-Revised Aphasia Quotient, CPIB = Communicative Participation Information Bank, CCRSA = Communication Confidence Rating Scale for Aphasia, ALA = Assessment for Living with Aphasia, VAMS = Visual Analogue Mood Scales. There was no skewed data for these measures.

#### Preliminary efficacy data

Due to the feasibility design of this study, it was intentionally underpowered for definitive efficacy testing. However, clinical outcomes were analysed to investigate preliminary efficacy using ANCOVAs to ascertain differences between Immediate and Delayed groups for each outcome measure at Time 2, controlling for Time 1 [60]. The results indicate that LUNA shows preliminary efficacy with 50% of measures (9/18) showing medium or large effect sizes (bolded in Table 6) for group differences at Time 2 once Time 1 was controlled for. Medium effect sizes were noted for all levels of discourse (number of narrative words, CIUs, complete and multi-clause utterances, clear reference chains), language (WAB-R AQ), and psychosocial state (VAMS). Large effect sizes were noted for one discourse level—% complete and % multi-clause utterances–and were also significantly different even with low power, indicating a proportionate increase in these narrative structures.

**Table 6:** Between group differences with effect sizes for each measure at T2.

	T2		
	Mean (SD) Immediate	Mean (SD) Delayed	ANCOVA F (df) p η_p_^2 ^
Narrative words:			
number	599.29 (388.13)	506.21 (524.31)	**F(1,25) = 2.49, p = 0.127,** η_**p**_^**2**^ = 0.091*
percentage	69.49 (13.47)	67.28 (14.39)	F(1,25) = 0.17, p = 0.736, η_p_^2^ = 0.005
per minute	45.44 (21.54)	58.27 (30.03)	F(1,25) = 1.14, p = 0.295, η_p_^2^ = 0.044
CIUs:			
number	532.00 (365.92)	442.71 (471.03)	**F(1,25) = 3.47, p = 0.074,** η_**p**_^**2**^ = 0.122*
percentage	64.16 (13.55)	63.23 (12.89)	F(1,25) = 0.42, p = 0.524, η_p_^2^ = 0.016
per minute	40.03 (20.68)	50.49 (27.33)	F(1,25) = 0.55, p = 0.466, η_p_^2^ = 0.021
Utterances:			
complete	54.64 (38.71)	40.71 (43.98)	**F(1,25) = 2.42, p = 0.132,** η_**p**_^**2**^ = 0.088*
% complete	66.85 (22.66)	53.18 (28.87)	**F(1,25) = 4.91, p = 0.036,** η_**p**_^**2**^ = 0.164**
multiclause	22.07 (18.74)	21.64 (26.55)	**F(1,25) = 2.18, p = 0.152,** η_**p**_^**2**^ = 0.080*
% multiclause	27.55 (18.58)	25.30 (20.74)	**F(1,25) = 6.30, p = 0.019,** η_**p**_^**2**^ = 0.201**
Predicate Argument Structure	1.82 (0.19)	1.69 (0.24)	F(1,25) = 0.71, p = 0.407, η_p_^2^ = 0.028
Story Grammar, number	4.14 (1.23)	4.07 (1.90)	F(1,25) = 0.09, p = 0.771, η_p_^2^ = 0.003
Reference chains, number of clear chains	13.71 (11.67)	7.85 (9.62)	**F(1,25) = 3.81, p = 0.063,** η_**p**_^**2**^ = 0.137*
Western Aphasia Battery-Revised AQ	77.86 (12.01)	72.48 (13.3)	**F(1,25) = 2.38, p = 0.135,** η_**p**_^**2**^ = 0.087*
Communicative Participation Information Bank	13.71 (4.00)	12.00 (5.46)	F(1,25) = 0.14, p = 0.708, η_p_^2^ = 0.006
Communication Confidence Rating Scale for Aphasia	29.64 (3.95)	27.50 (4.47)	F(1,25) = 1.00, p = 0.328, η_p_^2^ = 0.038
Assessment for Living with Aphasia	2.79 (0.55)	2.44 (0.50)	F(1,25) = 1.32, p = 0.262, η_p_^2^ = 0.050
Visual Analogue Mood Scales	6.89 (11.59)	15.39 (15.84)	**F(1,25) = 2.52, p = 0.125,** η_**p**_^**2**^ = 0.092*

**Bold text** indicates results with moderate to large effect sizes, where

** = large effect size (>0.14)

* = medium effect size (>0.06). Please note we have not adjusted for multiple comparisons because clinical outcomes in this feasibility study are considered preliminary only.

We additionally ran a non-parametric Wilcoxon on all the measures that the ANCOVA showed as having a **large effect size. We found that all narrative variables improve for the active group, and none for the control group. For WAB and VAMS the Wilcoxon results are not significant for either group (as per the ANCOVA). Note that the parametric effect sizes (as shown in the table) are needed here because calculating effect size cannot be reliably done for non-parametric analysis. Correlational analysis was additionally undertaken to explore which factors were associated with optimum response to the LUNA treatment, but there were no convincing patterns of predictors that would inform future studies or practice. See S1 File, for the detail.

#### Preliminary power calculation

Based on our medium WAB-R effect size of η_p_^2^ = 0.08 and above (equivalent F-effect size = 0.30), significant effects at α = 0.05 and 80% power = 0.8 would be detected by ANCOVA with a total sample size of 90 people (45 in each group; calculated using G*Power, [61].

### Safety

Adverse Events were logged and are reported by participant. Four participants (4/28, 14%) had a new health event. Two participants broke bones, one participant’s health deteriorated, and one participant had a further haemorrhage, a known risk factor within the stroke population [62]. These were unrelated to trial activity. Reports of distress were recorded by session and nine episodes were recorded across the 308 sessions in the trial (9/308, 3%). Episodes of distress were connected to the activities of the trial e.g., a participant became upset when asked to reflect on the impact of aphasia on their lives in the ALA assessment, and one episode was due to distress that the trial was finishing. Episodes were managed in accordance with an established protocol, and in discussion with the project manager.

## Discussion

Feasibility findings are positive across all aspects of recruitment, retention, adherence, missing data, treatment fidelity, and appropriateness of selected outcome measures (with one exception) and collectively support a future evaluation of LUNA in a definitive trial. Additionally, participants’ clinical outcome findings are promising for discourse, language, and psychosocial state; with particular beneficial treatment effect noted for discourse production at the sentence level. These findings are considered in turn below. ​ At 85% of those eligible, recruitment in the remote LUNA trial was more than double the average stroke trial [63], and other remote trials for aphasia such as the ‘Big Cactus’ study at 34% recruitment [64] and ‘TeleGain’ online groups at 10% recruitment [32]. The rate of recruitment was exceptional at 14 participants per month. Typically, aphasia recruitment rates are similar to stroke overall at 1–2 recruited per month [63–65]. This finding is most likely influenced by the pandemic, wherein it was estimated that nearly two thirds of SLT sessions were cancelled by services in the period from March-June 2020 [66], resulting in increased demand for SLT and general availability of participants with other life activities curtailed by the pandemic. Other explanations for this finding include the study being 1) largely non-restrictive inclusion criteria; 2) remotely delivered, enabling access to a wider pool of participants (supported by the wide geographical spread of resulting sample) and removing physical and transport barriers that often arise for this participant group; and 3) a *treatment* trial for chronic aphasia with waitlist-control design offering treatment to *all* participants, in the context of generally limited treatment provision for this group [67]. A weakness in the recruitment was the lack of diversity in the ethnicity, education level and socioeconomic status of participants. Possible reasons for this include the remote delivery creating digital access issues.

Retention was high which, similar to the reasons for high recruitment, may have been influenced by participant interest and availability in remote treatment from the convenience of home. It is also likely influenced by 1) trial length, wherein shorter studies have higher retention (e.g., exemplified by the difference in retention at the 19 weeks (98%) and 45 week (17%) follow up points in one study [68]); 2) provider involvement wherein SLT-delivered interventions usually have higher retention than self-directed interventions (e.g., ‘TeleGain’ [32] compared to ‘Big Cactus’ [64]); and 3) supportive trial practices namely upfront scheduling, participant-sensitive scheduling (considering individuals’ timetables/constraints), and appointment reminders [69, 70]. A further motivating factor may have been working in treatment on a personally chosen narrative.

Adherence findings were extremely positive with 87% of treatment sessions completed as scheduled, and a high proportion of participants completing most of the LUNA programme. Several factors may explain these findings. Firstly, as above, supportive trial practices enabled participants to attend assessment sessions at a convenient time (although for treatment, regular appointment slots were scheduled). Secondly, remote delivery both removes the physical barriers relating to mobility and geography that people with aphasia experience and affords convenience; and participants reflected these reasons in their acceptability interviews (manuscript in preparation). Thirdly, approximately half the sample considered themselves ‘confident’ or ‘very confident’ in using technology, and in using Zoom, on entry to the study, which may have mitigated the usual language and technological challenges of Zoom. Finally, findings suggest that participants were committed and motivated to complete the LUNA treatment.

Regarding participants’ clinical outcomes across the WAB-R AQ, CPIB, CCRSA, ALA, and VAMS-Sad, remarkably there were no missing data points, with all questions answered. Pre-emptive and sustained supportive trial practices during testing points are likely to explain this finding. Assessors developed a comprehensive ‘assessment checklist’ with a general framework which was then specified for each outcome measure, pre-empting assessor and participant needs in relation to the: environment (online and in participants’ own homes), equipment (internet, device, software, audio, visual), test material needed to complete assessment, test administration (guidance for assessors on preparing, instructions, stimuli, response, scoring), and evaluation (response requirements, performance). Assessors drew on guidance for remote delivery, and adaptations for remote participant response e.g., annotation and remote control. Assessors employed strategies to intentionally support participants and minimise challenge, dis-engagement, and error including (1) personalised approach (e.g., assessment packs were tailored to the device being used by each participant e.g., laptop or desktop vs iPads and Android tablets, so participants viewed guidance exactly as it appeared on their screens); (2) accessible communication, using visual supports for technology, and repetitive format to reduce cognitive demands; (3) attentiveness and flexibility e.g., monitoring fatigue and adjusting participant level of involvement required with technology where able; (4) transparency with participant and anyone in the home environment regarding privacy and assessment requirements; and (5) increased emphasis on managing distress and emotional engagement e.g., protocol for managing distress triggered by any assessment questions, and respecting participants’ preferences for privacy (especially relevant to some assessment questions). Such considered effort in this trial has proved beneficial for participant engagement and resultant data quality and will be replicated in the definitive trial.

Treatment fidelity is a core consideration when planning novel treatments [71], and was established as high in this trial [39] suggesting the time investment in creating a quality and comprehensive treatment manual and provider training were effective at enabling faithful delivery of the treatment. Additionally, the structured nature of sessions and structured order to the treatment programme delivery is likely to have contributed to the positive fidelity findings. Prospective development of the fidelity checklist with involvement [72] and activity logs [71] are strengths in fidelity evaluation, that were incorporated in this trial. The fidelity data revealed some areas for future attention, including further scrutiny of missing elements in SSLT led sessions. The lowest scoring aspect of the treatment (Item 8 on the fidelity checklist) related to how both ‘story’ and ‘non-story’ targets are incorporated in LUNA treatment. The manual specifies that treatment stimuli (words, sentences, story components) should be chosen to include both ‘story’ items and ‘non-story’ items to promote generalisation of gains beyond the treated story. ‘Story’ items are treatment targets which will eventually be used in the treated story (i.e. story words; story sentences; story macrostructure elements) and ‘non-story’ items are treatment targets that are not intended for use in the treated story but which are related (either syntactically, semantically, or structurally) to those targets that are intended to be used in the story.

Following published guidance [73], a traffic light system of progression criteria for feasibility outcomes for a trial such as this was suggested as: feasible if >35% of those eligible are recruited (green), with <20% not feasible (red). Retention is feasible if >85% of participants are retained at follow up (green), with <65% not feasible (red). Treatment fidelity is considered feasible if >75% (green), and not feasible if <50% (red). As such, remote LUNA meets all the criteria proposed to progress to a definitive trial.

We acknowledge that the feasibility outcomes for this remote LUNA trial should be considered cautiously with respect to evaluating LUNA in a future face-to-face trial. It is encouraging that such positive findings were achieved despite the barriers of working online, and against the problematic background of the pandemic. We note however that retention and adherence findings are supported by eliminating participant travel and the fact that so many other services were curtailed during the pandemic. More consideration of supportive trial practices for this participant group is needed if delivery reverts to in person.

Blinding is an important marker of quality in trials as it reduces bias [74, 75]. However, few studies evaluate it or report whether it was maintained [76]. Assessors were unblinded for 25% of participants. In some instances, it may be that this was because a rapport existed with the assessor so they were potentially seen as a trusted person e.g., when a participant could not access Zoom for their treatment session, they called for technological support from the Assessor rather than the SLT, SSLT or Project Manager. Further consideration is needed in future to avoid such instances from occurring in a definitive trial.

Although not powered to provide conclusions about clinical efficacy, effect sizes can indicate where a future definitive trial may show treatment effect. LUNA’s preliminary efficacy findings are positive for discourse (at all three levels of language), language functioning, and psychosocial state (specifically mood) with medium effect sizes; as well as demonstrating treatment effect for utterance level discourse (large effect sizes, and significantly greater percentages of complex and multi-clause sentences in Immediate participants’ personal narratives, compared to Delayed participants). Additionally, it was encouraging to see preliminary efficacy for numbers of CIUs which is the most frequently reported discourse indicator [13].

These findings are likely explained by the existing but limited evidence base indicating that multi-level treatment provokes multi-level change [13]. There is also existing evidence of a relationship between discourse and overall language, where studies of other discourse treatments such as scripting have also shown benefits for overall language functioning [77, 78]. Compared to other multi-level treatments, these findings suggest that LUNA has the potential to offer more comprehensive discourse outcomes. Hoover and colleagues [18] describe multi-level treatment activities with 12 participants, reporting significant gains at the utterance and discourse macrostructure levels but not for words; Whitworth [20] reports single-case evidence for multi-level treatment producing gains across utterance and discourse macrostructure levels (and, for one of the two participants, also at word level); and Whitworth and colleagues [19] report within-group pre/post gains across all three levels for 14 participants but, at the group level, these gains did not differ significantly from the control group. The positive effect size findings from remote LUNA represent promising potential for beneficial group gains at all 3 levels of language.

Although there was a medium effect size noted for the VAMS outcome measure of mood, it showed ceiling effects with more than 15% of the sample scoring the maximum possible score of 0 at each of the timepoints [79]. Such a finding might raise concerns about content validity and responsiveness suggesting reconsideration of this outcome measure for inclusion in definitive trial testing. Of note is the choice of the VAMS-Sad scale, meaning that mood was evaluate with a single scalar question. An outcome measure with more questions, interrogating different aspects mood might be beneficial in a future trial.

There was no indication of preliminary efficacy for other measures of psychosocial state, namely communication confidence, communicative participation, and aphasia-related quality of life. Psychosocial state has previously been minimally measured as an outcome from discourse treatment [13] and as such deserves continued attention in the future. There are three possible explanations for this finding. Firstly, LUNA treatment may not be sufficiently potent to improve psychosocial state. Secondly, the outcome measures may not be sensitive enough, and reviewing the additional qualitative data will help guide future outcome measures consideration. Thirdly, and most likely, the data was collected throughout the COVID-19 pandemic, through various lockdowns and release, and this context is highly likely to have affected how participants responded to questions in the psychological state measures. As such, it is not possible to make decisions about psychosocial state outcome measure selection for a future trial based on these findings.

The analysis used a novel protocol for measuring language using a person’s life stories. This measure has benefits: it is based on a personal story so is likely to reflect change that is meaningful for the individuals involved; it has shown sensitivity in that several metrics from the analysis showed significant group differences following treatment and/or large effect sizes. However, there are concerns about tester burden, in that the story must be transcribed and analysed. Further developmental work could seek to find ways to make discourse analysis more efficient, and to further explore the psychometric properties of measures for personal narrative discourse.

### Limitations

Some limitations are noted. Firstly, the sample recruited to this remote feasibility trial is not typical of the wider stroke and aphasia population and future studies should aim to recruit a more representative sample. With a mean age of 60 years, this sample was younger than both a national sample, a mean age of 78 years [80] and a London sample of 68.9 years [81]. Additionally, both London and national samples have more ethnic diversity reporting 56% and 95.7% white participants respectively, compared to the 100% white sample in the remote LUNA study [81, 82]. Secondly, measuring change in spoken discourse is a challenging undertaking, as there are numerous metrics used in the research field and their psychometric properties are generally not well established [11, 83]. To address this problem, this study employed: (1) traditional discourse metrics used in many research studies e.g., number of CIUs; (2) discourse metrics with proven psychometric properties of reliability and validity [40, 41, 83]; and (3) a novel word-level metric of narrative words intended to act as a comparator for CIUs to explore the possibility that it would be more clinically feasible. Further analysis not reported here does not support the notion that the narrative words measure is a straightforward alternative to CIUs, and further research is needed with any novel measures subjected to traditional psychometric testing. Thirdly, the LUNA Discourse Analysis Protocol was created for this study and has some, not insignificant, assessor burden with analysis of each narrative at each time point taking approximately three hours. However, this represents the time for the research version of the LUNA discourse analysis protocol and the intention is to reduce this protocol in the future for clinical implementation. Finally, most of the clinical outcome measures were not validated for online delivery, except for WAB-R which has demonstrated equivalence [84], but differences in outcomes between face-to-face and online delivery of the Boston Naming Test demonstrate this cannot be assumed [85].

### Future implications

This study’s findings meet the set criteria for progression to definitive trial testing, in the context of remote treatment delivery. LUNA was co-designed as a face-to-face intervention but delivered online due to the COVID-19 pandemic, and the positive feasibility findings presented here are of remote LUNA. Future studies could consider a similar study of face-to-face delivery, compare face-to-face with remote delivery, or co-design a hybrid delivery model.

LUNA appears to have potential clinical value because of its multi-level language focus, personalised narrative approach, and emphasis on metalinguistic and metacognitive skills which translate well towards self-management during and following treatment. The original co-design of LUNA with providers and recipients of SLT [22] also strengthens LUNA’s applicability and relevance to the treatment of people with chronic aphasia in UK clinical settings. This approach serves as a good example for the development of further interventions seeking to embed co-design, salience, and authentic, functional language change.

## Conclusions

The remote LUNA trial satisfied all feasibility progression criteria for stroke trials in trial recruitment, trial retention, and treatment fidelity. High levels of participant adherence to treatment sessions and completion, and low counts of missing data suggest remote LUNA is acceptable. Preliminary efficacy is indicated for all three levels of discourse, and overall language functioning, suggesting that it is worth exploring the clinical efficacy and cost-effectiveness of LUNA in a future definitive trial.

## Supporting information

S1 Checklist LUNA Template for Intervention Description and Replication (TIDieR). (DOCX)

S2 Checklist CONSORT 2010 checklist of information to include when reporting a pilot or feasibility trial. (DOCX)

S1 Appendix LUNA discourse metrics. (DOCX)

S1 File Additional correlational analysis for the LUNA clinical outcome measures. (DOCX)

S1 Protocol LUNA trial protocol. (DOCX)

S1 Dataset LUNA dataset. (SAV)
