# Supplementary material for: A feasibility randomised waitlist-controlled trial of a personalised multi-level language treatment for people with aphasia: The remote LUNA study
Source: PLoS One. 2024 Jun 14;19(6):e0304385. doi: 10.1371/journal.pone.0304385 (PMC11178191; doi:10.1371/journal.pone.0304385)
Supplement: S1 Checklist — (DOCX) [file pone.0304385.s001.docx]

## **S1 Checklist**

## **LUNA Template for Intervention Description and Replication (TIDieR)**

For full study protocol, please see <https://clinicaltrials.gov/study/NCT05847023>

1. **Name of intervention**

Language Underpins Narrative in Aphasia (LUNA)

1. **Rationale, theory, or goal of the elements essential to the intervention**

LUNA is a multi-level intervention with a strong semantic (meaning) focus improving a

personal narrative. Essential elements include:

- LUNA seeks to make the person’s story *richer, more meaningful, more complete, make more sense, and flow more*. These key words are core to LUNA’s treatment and serve as reminder to the person and therapist that this is the purpose of treatment.
- Eliciting, analysing and treating the personal narrative generating entirely personalized treated stimuli which by virtue of personal relevance should motivate the individual to engage in rehabilitation, and should also feature with higher frequency in the person’s everyday life thereby yielding more practice opportunities outside the treatment session.
- Analysing and treatment word, utterance and discourse levels; existing research shows that single or sequential focus on these levels does not generalise to everyday language and different approaches need to be considered in novel interventions.
- A meta-linguistic and meta-cognitive approach (‘meta-awareness’) is taken throughout, enabling the person to understand their language and discourse strengths and difficulties, and know how to treatment aims to improve discourse performance, and thus how to actively self-manage within and beyond the treatment session.
- Semantic feature analysis treatment is adopted for the treatment of single nouns at the word level, and mapping therapy at the utterance level. Story grammar and reference chains are targeted at the macrostructure level.
- The partnership approach to working collaboratively is reflected throughout with respect for the person’s perspective and preference considered at all times. Collaborative engagement in this treatment engenders an adult-learning style approach and motivates the learner.

1. **Materials**

Written transcript of the participant’s personal narrative and written activities (both

available via screen share) following the treatment manual for each session. Participants’

personal technological devices e.g., smartphone or iPad may be used at participants’

request. The LUNA treatment manual will be available following completion of the study.

1. **Procedures**

The LUNA treatment programme comprises (1) joint review of the participant’s personal

narrative (written transcript of spoken narrative) to understand strengths and difficulties

and agree treatment goals; (2) session activities targeting word, utterance and discourse

levels of narrative incorporating semantic feature analysis therapy, mapping therapy, and

discourse treatment of story grammar, cohesion, and coherence; (3) explanations and

reflective tasks that enable meta-linguistic and meta-cognitive understanding of the

participant’s profile and the LUNA treatment; and (4) activities for self-management

outside the clinical session. E-Handouts written in a communication accessible manner (and

made available via screen share and email and post if needed) will support the participant

during and outside sessions to make progress towards goals.

1. **Intervention professional**

50% of the sessions will be delivered by a qualified speech and language therapist trained

in the LUNA programme. The remaining 50% of the sessions will be delivered by a

therapy assistant (student speech and language therapist also trained in the LUNA

programme) and delivered under the supervision of qualified therapists.

1. **Intervention delivery**

Intervention is delivered remotely via Zoom and individually, for the majority of the

sessions. A small proportion of sessions (no more than 15%) may be delivered in a pair or

small group and is dependent on participant preference and interest – this will occur

remotely in Zoom and may use the Breakout Rooms facility available.

1. **Delivery location**

Treatment delivered remotely, with the participant and treating therapist both working

from their own home.

1. **Intensity and Dose**

Two sessions (60 minutes each) a week for 10 weeks.

1. **Tailoring**

The stimuli/targets used in this study are derived from each participant’s personal

narrative. As such, the targets are entirely personalized. Despite this level of

personalization, elements across the intervention are intended to be held relatively constant e.g., number of sessions at word, utterance and discourse levels. This includes prescribed therapy activities within the two sessions per week and advised activities for self-management of practice outside the session. Participants and therapists will agree choice of

self-management activities from a list of recommended activities.

1. **Modifications**

Currently not applicable.

1. **Planned treatment fidelity**

Therapists and therapy assistants will be trained in an intervention manual. 10% of therapy

sessions will be video-recorded and assessed by trained raters for adherence to the

treatment manual via a treatment fidelity checklist. Therapists and therapy assistants will

self-assess their adherence by completing the treatment fidelity checklist at the end of each

therapy session.

1. **Actual treatment fidelity**

The LUNA treatment was delivered as intended, with high adherence to the manual. 91.6% of items were marked as present (616/672).
