# Supplementary material for: A feasibility randomised waitlist-controlled trial of a personalised multi-level language treatment for people with aphasia: The remote LUNA study
Source: PLoS One. 2024 Jun 14;19(6):e0304385. doi: 10.1371/journal.pone.0304385 (PMC11178191; doi:10.1371/journal.pone.0304385)
Supplement: S1 Appendix — (DOCX) [file pone.0304385.s003.docx]

## **S2 Appendix**

## **LUNA Discourse Metrics**

Word-Level Indicators for Assessment of Personal Narratives

- Correct Information Units (CIUs)
- CIUs are “words that are intelligible in context, accurate in relation to the picture(s) or topic, and relevant to and informative about the content of the picture(s) or the topic” (Nicholas & Brookshire, 1993, p. 348).
- We counted them and then reported them as an overall total for the discourse (#CIUs), as a percentage of the total number of all words (%CIUs), and as measure of efficiency (CIUs per minute).
- Narrative words
- Narrative words are a similar construct to CIUs, but their definition is different and is broader. There is more than one definition of narrative words in the research literature, but we recommend Saffran et al. (1989) for the most widely-used operational definition. For the LUNA package we created a simplified definition (based on Saffran et al. 1989) but encompassing more words), as follows: narrative words are the words that remain in a discourse once the following have been removed:
- Non-linguistic fillers (e.g., *er*, *um*, *uh*)
- Linguistic fillers (e.g., *yeah’,* ‘*well’* or *‘so’*)
- False starts (e.g.,  *diff- er different um different from oh we were individuals*,)
- Repetitions (e.g,. *a boat .. a boat er ..a big boat*)
- Extra-narrative content (e.g., commentary about performance on the task, such as “*I can’t think of the word.*”)
- We counted narrative words and then reported this count as an overall total (#NWs), as a percentage of the total number of all words (%NWs), and as measure of efficiency (NWs per minute).

Sentence-Level Indicators for Assessment of Personal Narratives

- Complete utterances (C-Units)
- Utterances are defined in LUNA as a group of words which are grammatically connected to each other (but not to any words outside the utterance). Saffran et al. (1989) using a similar definition, advocated the combined use of syntactic, semantic and prosodic indicators to make a decision about utterance boundaries. We followed this guidance.
- C-Unit: a main clause and all its dependent clauses.
- The distinction between dependent and independent clauses is crucial in finding utterance boundaries, which means that it is also important to distinguish the different types of conjunctions used to link clauses. There are coordinating conjunctions and subordinating conjunctions. The segmenting rule is to:
- Separate utterances that are linked by a co-ordinating conjunction (into two utterances).
- Keep together (as a single utterance) clauses that are joined by a subordinating conjunction.

There is disagreement in the literature about which conjunctions are subordinating conjunctions. We took a narrow approach and included only “and”, “and then”, and “or” in this category.

- Once a transcript had been divided into utterances, we coded each utterance as either *complete* or *incomplete*. The decision about completeness is both syntactic and semantic, with most emphasis on the syntax. Utterances are *incomplete* when they omit a key information-carrying word (e.g., a verb, noun or key preposition) or phrase (e.g., a verb phrase, noun phrase or key prepositional phrase). *Complete* utterances are those which appear to contain all necessary words OR those which omit only minor words (e.g., function words) such as determiners, auxiliary verbs, and less important prepositions (i.e., not those required by a verb).
- We counted the total number of utterances in a discourse (# utterances); counting the number of complete and incomplete utterances (#complete, #incomplete); and calculated the percentage of complete utterances (% complete). This yielded a measure of completeness.
- Multiclause utterances
- We coded each utterance in terms of its complexity. We coded in a binary way (i.e. single-clause vs multi-clause). It might also be useful to code utterances in terms of how many dependent (i.e., subordinate or relative) clauses are combined with a main clause.
- We counted single- versus multi-clause utterances (#single, #multi) and calculated the percentage of multi-clause utterances in a discourse (% multi). This yielded a measure of syntactic complexity.
- Predicate-argument structure (PAS)
- We examined the PAS of each clause within an utterance.
- A clause is defined as a syntactic unit containing both a noun phrase and a verb phrase; and as a semantic unit containing both a predicate. (a verb or verb group) and arguments.
- Arguments are the phrases needed to complete the core meaning of a predicate. For example, the verb ‘throw’ requires [someone] to throw [something]; and the verb ‘put’ requires [someone] to put [something] [somewhere]. It helps to think about sentence elements that you would embody if you were to gesture a sentence’s meaning – each embodied element would be an argument.
- Non-arguments (or adjuncts) are phrases containing semantic information that is not core to a verb’s meaning such as place, time, measurement, or accompaniment. Note that these would not be part of a gesture or the predicate meaning.
- We counted the number of predicates and arguments in the discourse (# predicates, # arguments) and then also calculated a mean PAS score (= # arguments/# predicates). This yielded a measure relating to semantic complexity.

Macrostructure-Level Indicators for Assessment of Personal Narratives

- Story grammar
- Story grammar is a macrostructure (or superstructure) measure that identifies story elements from a predetermined set which are expected to occur at either the beginning, middle or end of a discourse. There are various versions of the set of story grammar elements in the aphasia literature. In LUNA we use the following nine elements:
- Beginning – abstract, time, location, participants
- Middle – initiating/complicating event, events
- End – evaluation, result/resolution, coda/conclusion.
- We marked each of the nine elements as either present or absent, to provide a measure of overall superstructure skill and to identify targets for treatment (#SG elements present).  This simple present/absent scoring may not be sensitive enough to measure post-treatment change, however, and so more work needs to be done on the psychometrics of this analysis before a decision can be made about its use in a definitive trial.
- Reference chains
- Reference chains are chains of words in disparate places across a discourse that refer to the same person, place or thing. We confined identification to only those chains where the first referent was a noun (or noun phrase) and the others were pronouns.
- We counted clear versus unclear reference chains (#clear, #unclear) which yielded a measure of cohesion.
