# Supplementary material for: A feasibility randomised waitlist-controlled trial of a personalised multi-level language treatment for people with aphasia: The remote LUNA study
Source: PLoS One. 2024 Jun 14;19(6):e0304385. doi: 10.1371/journal.pone.0304385 (PMC11178191; doi:10.1371/journal.pone.0304385)
Supplement: S1 File — (DOCX) [file pone.0304385.s004.docx]

## **S3 Correlational Analysis**

**Additional correlational analysis for the LUNA clinical outcome measures**

Overall, there were no convincing patterns of association to inform a future study or practice – see table 7, below. However, there were some sporadic effects to note: participant age, comprehension ability, and stroke severity were moderately associated with change, but only for the two word-level discourse measures (narrative words and CIUs) rather than changes in discourse more broadly: age was also associated change on the mood score; and two of the baseline language scores (% multiclause utterances and WAB Spontaneous Speech score) were associated with improved WAB AQ.

**Table 7: Correlational analysis**

|  | | | Narrative words  change | CIUs  change | Complete percentage utterances  change | Multiclause percentage utterances  change | Clear reference chains  change | | WAB AQ  change | VAMS score  change |
| --- | --- | --- | --- | --- | --- | --- | --- | --- | --- | --- |
| **Demographic factors** | | | | | | | | | | |
| Age at recruitment | | Correlation | **-.386*** | **-.416*** | -.239 | .081 | -.173 | | .012 | **-.395*** |
|  |  | Sig. (2-tailed) | .042 | .028 | .221 | .683 | .388 | | .950 | .037 |
|  |  | N | 28 | 28 | 28 | 28 | 27 | | 28 | 28 |
| Aphasia severity | | Correlation | **.381*** | **.440*** | .202 | .263 | .207 | | -.221 | .103 |
|  |  | Sig. (2-tailed) | .045 | .019 | .302 | .176 | .300 | | .257 | .602 |
|  |  | N | 28 | 28 | 28 | 28 | 27 | | 28 | 28 |
| Months post stroke† | | Correlation | -.034 | -.060 | -.109 | -.172 | .074 | | **.331** | .007 |
|  |  | Sig. (2-tailed) | .864 | .763 | .583 | .380 | .712 | | .085 | .973 |
|  |  | N | 28 | 28 | 28 | 28 | 27 | | 28 | 28 |
| **Baseline language and cognition factors** | | | | | | | | | | |
| Total words† | Correlation | | -.074 | -.049 | .215 | **.306** | .245 | | -.290 | -.147 |
|  | Sig. (2-tailed) | | .709 | .803 | .272 | .113 | .218 | | .134 | .454 |
|  | N | | 28 | 28 | 28 | 28 | 27 | | 28 | 28 |
| Narrative words† | Correlation | | -.027 | -.003 | .238 | **.314** | .248 | | -.297 | -.128 |
|  | Sig. (2-tailed) | | .892 | .989 | .223 | .104 | .212 | | .125 | .515 |
|  | N | | 28 | 28 | 28 | 28 | 27 | | 28 | 28 |
| Percentage of complete utterances | Correlation | | .237 | .262 | -.011 | .093 | .128 | | -.299 | -.122 |
|  | Sig. (2-tailed) | | .224 | .178 | .957 | .640 | .523 | | .123 | .535 |
|  | N | | 28 | 28 | 28 | 28 | 27 | | 28 | 28 |
| Percentage of multiclause utterances | Correlation | | .218 | .244 | .043 | .197 | .274 | | **-.550**** | .100 |
|  | Sig. (2-tailed) | | .265 | .211 | .829 | .314 | .166 | | .002 | .614 |
|  | N | | 28 | 28 | 28 | 28 | 27 | | 28 | 28 |
| Overall language (WAB AQ) | Correlation | | **.349** | **.397*** | .257 | .224 | .166 | | **-.302** | .085 |
|  | Sig. (2-tailed) | | .068 | .037 | .187 | .253 | .408 | | .119 | .668 |
|  | N | | 28 | 28 | 28 | 28 | 27 | | 28 | 28 |
| Spontaneous speech score (WAB) | Correlation | | **.330** | **.382*** | .179 | .280 | .257 | | **-.393*** | .007 |
|  | Sig. (2-tailed) | | .087 | .045 | .361 | .149 | .195 | | .038 | .972 |
|  | N | | 28 | 28 | 28 | 28 | 27 | | 28 | 28 |
| Comprehension score (WAB) | Correlation | | **.457*** | **.477*** | .180 | .055 | .169 | | -.225 | .193 |
|  | Sig. (2-tailed) | | .015 | .010 | .360 | .781 | .400 | | .249 | .326 |
|  | N | | 28 | 28 | 28 | 28 | 27 | | 28 | 28 |
| Cognition (RCPM)† | Correlation | | .168 | .188 | -.042 | -.001 | | -.238 | -.259 | .088 |
|  | Sig. (2-tailed) | | .392 | .337 | .831 | .997 | | .231 | .183 | .656 |
|  | N | | 28 | 28 | 28 | 28 | | 27 | 28 | 28 |

†Spearman correlation used. WAB=Western Aphasia Battery, AQ=Aphasia quotient, RCPM=Ravens Coloured Progressive Matrices. *significant at p<0,05, **significant at p<0.01. **Bold** indicates a moderate value and **bold and shaded** indicates a significant value
