# Supplementary material for: A feasibility randomised waitlist-controlled trial of a personalised multi-level language treatment for people with aphasia: The remote LUNA study
Source: PLoS One. 2024 Jun 14;19(6):e0304385. doi: 10.1371/journal.pone.0304385 (PMC11178191; doi:10.1371/journal.pone.0304385)
Supplement: S1 Protocol — (DOCX) [file pone.0304385.s005.docx]

## **S6 Trial Protocol**

**Language Underpins Narrative in Aphasia (LUNA): A novel discourse treatment for people with aphasia: Protocol for a feasibility randomised controlled trial testing feasibility, acceptability, initial efficacy and fidelity**

**Version 2 22/05/2020 -amended in response to COVID-19 interruption**

**Aims**

This study will test feasibility of LUNA in terms of participant recruitment and retention, and adherence throughout the study (assessment and treatment sessions). We will explore participant acceptability of LUNA and project procedures (satisfaction, perceived benefits, assessment and treatment burden) in semi-structured individual interviews with participants. We will investigate initial efficacy by comparing outcomes on a range of measures across participants who have and have not received LUNA intervention. We will assess treatment fidelity by reviewing a sample of videotaped sessions to check they were delivered as intended by the treatment manual. The study will also test the feasibility and acceptability of remote assessment and treatment delivery. For example, recruitment, retention and compliance data will attempt to identify losses due to technological factors; and interview questions will explicitly probe the experience of using video-conferencing technology. Indicative outcome data will be for remote (not face-to-face) LUNA.

**Methods**

**Design**

The study is a single-blind, waitlist, randomised controlled trial of LUNA for people with chronic post-stroke aphasia. Participants will complete assessments at three time points: T1 (weeks 1&2), T2 (weeks 13 & 14) and T3 (weeks 25 & 26). Following T1, participants will be randomised to an immediate or delayed condition. Those in the immediate condition will receive LUNA therapy between T1 and T2. Those in the delayed condition will receive LUNA therapy between T2 and T3. All participants will be invited to participate in in-depth individual semi-structured interviews post-therapy to explore their experiences of taking part in the study.

In line with our selection criteria, participants will not be receiving any other speech and language therapy during the study. Owing to COVID 19, it is anticipated that most participants will not have access to usual care (attendance at stroke groups). Some may access on-line supports, for example provided by Aphasia Re-Connect. Use of such services is not an exclusion criterion. Use of other services will be explored in the interviews.

We will follow the CONSORT 2010 extension statement to randomised feasibility trials in planning, conducting and reporting on the study <https://www.bmj.com/content/355/bmj.i5239>.

**Ethical approval**

Ethical approval to conduct the study was granted by the School of Health Sciences Research Ethics Committee, City, University of London (ETH1920-0210 on 04/02/2020). The trial sponsor is City, University of London, and the study is funded by the Stroke Association (TSA2017/01). Our funder has formally approved the adapted study (20/05/2020).

**Participants**

***Setting***

Participants will be identified from community stroke support groups across England. Co-ordinators of the stroke groups will be emailed and invited to refer potential participants. The study will also accept self-referrals, from any person with aphasia living in the UK.

***Remote Data Collection***

All recruitment, assessment, treatment and interview sessions will be conducted online using Zoom video conferencing technology. This platform was recommended by our aphasia advisory group and has been used in previous aphasia studies (Dekhtyar et al, 2020). It has been approved for use by our institution (City, University of London) and is widely used by NHS and voluntary sector services. A number of features make this platform suitable for aphasia intervention, including low language demands, a whiteboard facility and screen sharing.

***Inclusion and exclusion criteria***

Inclusion: Participants must be adults (18+ years); diagnosis of ischaemic or haemorrhagic stroke; and have aphasia due to a stroke that occurred at least 12 months prior to recruitment. Participants must be literate and fluent users of English prior to their stroke (self-reported). They must have adequate hearing and vision with aids and glasses, e.g. in order to see pictorial and written assessment and therapy materials. Participants must have access to a computer or tablet and an internet connection. They must be able to download and access Zoom, either independently or with the support of a friend/family member who is living with them.

Exclusion: Participants must not be receiving speech and language therapy elsewhere for the duration of the study. They must not be participating in any other aphasia treatment research project for the duration of the study. Usual stroke supports, for example provided by the voluntary sector, can proceed, although these are likely to be curtailed owing to COVID-19. They must not have severe aphasia, defined as a score of 7 or less on the receptive domains (auditory and reading) and 7 or less on the expressive domains (speaking and writing) of the Frenchay Aphasia Screening Test (FAST: Enderby et al., 1986; unlikely to benefit from treatment). They must not have a secondary cognitive diagnosis such as dementia. This will be established via self-report and/or the confirmation of the referring group co-ordinator and/or by expert clinical judgment of research project staff (experienced therapists).

**Recruitment and consent processes**

Participants will be recruited from voluntary sector community stroke support groups or via self-referral. Researchers will make email and phone contact with group coordinators. Many also attended a project information event in February 2020 held at the university. Members of the research team will provide information to group coordinators and to any individuals enquiring about the project. Coordinators will make direct referrals of individuals (with their Permission) to the research team. The study will also be advertised through the project website via a blog posting (<https://blogs.city.ac.uk/luna>) and social media (project twitter handle @LUNA_Aphasia) will be used to draw attention to the blog posting. Those who are interested in the project will have a 1:1 conversation with a member of the research team to discuss potential eligibility further. A member of the research team will carry out the information and consent process with individuals via Zoom.

We acknowledge that this project is not appropriate for individuals with severe aphasia. We endeavour to attract and screen only individuals with aphasia who are likely to have mild and moderate impairments in their language abilities; and we will not actively advertise the project to individuals with severe aphasia. Firstly, we will manage this sensitively in our communications with community stroke and aphasia group coordinators so that they understand clearly who the project is likely to benefit and who it will not. That is, individuals who find it difficult to say and understand single words would not benefit. Secondly, we will communicate clearly with potentially eligible participants by describing in lay language the kind of language ability profiles for whom this project may benefit and those for whom it won’t. For example, “if you can say and understand single words and sentences most of the time, then this project is for you”; “if saying any words or understanding words is hard, this project is not for you”.

a) Consent will be obtained primarily by the SLT researcher on the project (Swinburn) but may also be supplemented by members of the research team (Cruice, Marshall, Dipper, Botting) who are based at City and will be involved in recruitment activities as appropriate.

b) Participants will sign 2 copies of the consent form and will keep one.

c) Participants will receive the PIS prior to contact with the researcher. These will be posted and emailed to group coordinators (whose contact details are publicly available on the Stroke Association website). They will identify possible participants from their stroke group and forward the information to them. Individuals who express an interest will be referred by co-ordinators to LUNA. The PIS will also be emailed/posted directly to people with aphasia who make a self-referral.

Interested individuals will meet with a research team member 1:1 (or with family and/or significant other present if preferred by the person with aphasia). The meeting will take place over Zoom. The researcher will go through the PIS (reading it aloud), answer any questions about it, and check the individual’s understanding of the content as well the implications for them. The researcher will go through a simple screening interview and will administer the Frenchay Aphasia Screening Test (Enderby et al., 1986) to check eligibility (project is not suitable for people with severe aphasia). Those who are eligible and interested will be emailed the Consent Form. They will be asked to sign this before their next appointment which will occur at least 24 hours later. The Consent Form can be scanned or posted back to the LUNA team. Participants who are able may also wish to use the annotate function in Zoom to tick and sign the Consent Form which will be saved in electronic format.

d) Participants will have at least 24 hours between receiving information about the study and signing the Consent Form.

Informed consent is recognised as an important matter in this study. PWA do not have cognitive impairment and do not lack capacity as a result of their stroke and aphasia; however, informed consent is mediated through communication (reading printed information, understanding verbal explanations, speaking to question aspects and comment) and as such is more challenging with a language impairment. We have addressed this issue by providing information in an accessible format, following national and international guidance (including Pearl & Cruice, 2017). Those in the research and treating team are qualified staff who are experienced in communicating with PWA and recruiting individuals, and all new staff involved will receive training in supporting communication so that participants can fully engage throughout the whole research period. The project team has expertise in working remotely with people who have aphasia (see Woolf et al, 2016; Cruice et al, under-review).

**Randomisation**

Individuals will be randomized to the immediate or delayed condition by an independent person, blinded to screening results. Participants will be recruited in two cohorts of 12 participants each, starting at different time points so that there is appropriate staffing for the length of the project.

**Blinding**

Participants, the project manager, research speech therapists, the qualitative researcher, and the joint principal investigators will be aware of group allocation. However, the assessors (research assistants) who conduct the language, discourse and psychosocial assessments at all time points will be blinded to group allocation; as well the broader research team (co-investigators). Participants will be requested not to reveal their group allocation to staff during visits and will be reminded of this prior to the visit and at the start of the visit. Visits will be organised by the project manager, and research assistants will have no access to participant files or details. If a research assessor becomes unblinded, this will be reported, and subsequent visits carried out by another assessor. Near misses will also be reported. A log will be kept of all instances of unblinding and near misses, as well as the reason for the unblinding.

**Intervention**

LUNA intervention comprises 20hrs of treatment, 2 sessions per week of 60 minutes each, for 10 weeks. All sessions will be delivered over Zoom. One session is delivered by a qualified speech and language therapist; the other session is delivered by an assistant (student or volunteer) following guidance and under distance supervision. Both therapist and assistant will receive LUNA training. This model of delivery reflects the intended future implementation model in the NHS community setting and was decided by the LUNA Advisory Group. The treatment is specified in the TIDIER checklist (see end of document). In brief, it aims to improve spoken discourse production, using personal narratives as assessment and treatment stimulate, and by integrating word, sentence and discourse level tasks. Treatment is manualised and has been codesigned with key stakeholders (providers and users).

**Outcomes**

Clinical assessments at all three time periods will be conducted by individuals separate from the treating staff who will be blinded to group allocation. All sessions will be delivered over Zoom. Discourse (two personal narratives told at each time point) will be elicited and be analysed following the LUNA protocol. Analysers will be separate from the treatment staff and will also be blinded to group allocation. Interviews will be conducted by an individual trained in qualitative methods and separate from the treating staff. Interviews will follow a topic guide, and also include the 4 item Session Rating Scale as rated by the participant (which invites them to reflect on respect and understanding, relevance of the goals and topics, client-practitioner fit, and overall alliance). Specific questions will also explore the use of video-conferencing technology, for both assessment and therapy, and the degree to which this might have affected treatment acceptability and outcomes. Interviews will be conducted at post-therapy only and in a 3rd session separate from the assessment sessions. A random sample of 10% of treatment sessions will be recorded and observed and checked for adherence to treatment manual and conducted by individuals separate to the treating staff. Recording will be via the Zoom screen recording facility. Participants will give consent for this recording, both on the consent form and (verbally) prior to each recorded session. They will be advised on strategies that protect their privacy (such as background blurring/ replacement).

Data will comprise counts extracted from the screening, recruitment and data collection period (for recruitment (eligibility and consent rates), retention, adherence etc). Data will also comprise qualitative semi-structured interview responses from participants which will be subjected to thematic analysis (Framework). Data will also comprise discourse scores from personal narratives, language assessment scores, and psychosocial questionnaire scores. Assessments and questionnaires have been chosen on the basis that they are psychometrically robust and tested with an aphasic population and have been trialled with 4 PWA on our co-design and advisory group and deemed acceptable. Finally, data will also comprise essential elements deemed to be present or absent from recorded treatment sessions; intra- and inter-rater reliability will also be checked; findings will be assessed using percent agreement across the two raters.

**Primary endpoints**

1. *Feasibility of recruitment and retention to the trial*: Data will comprise counts/ proportion of those who express interest, are screened and deemed eligible, those who consent, attrition and reasons for attrition if known
2. *Acceptability of the intervention to participants*: Adherence data will be collected (i.e. number/proportion of treatment sessions attended; reasons for non-attendance). Interview data will illuminate participants’ views about the intervention together with scores on the Session Rating Scale (4 items completed within the qualitative interview)
3. *Acceptability of research procedures and outcome measures*: Data will comprise attrition rates and rates of missing data; and qualitative participant interview data on the study experience and procedures

**Secondary endpoints**

1. *Appropriateness of outcome measures*: includes level of variability of scores; missing data; floor and ceiling effects; whether scale constructs align with concepts described by participants during in-depth interviews; and participant in-depth interview on acceptability of measures
2. *Estimating sample size*: based on means and standard deviations and effect sizes of the proposed primary clinical outcome measures (linguistic outcomes, see below) and retention rates.
3. *Assessing treatment fidelity processes*: includes acceptability of fidelity checking processes to participants; utility and reliability of fidelity checklist; extent to which treatment is delivered as intended (as prescribed in the therapy manual)

***Outcome Measures***

All proposed assessments have good psychometric properties. Most were developed for people with aphasia and all have been used in previous aphasia therapy studies. They have also been recently reviewed and discussed by our advisory group of people with aphasia and speech therapists (meeting 05/11/2019). Currently the assessments in total take 2 hours and 20 minutes. In combination with telling the two narratives (15 mins each) and the demographic questions (<10 mins) there is ample time within the proposed 4 hours at each time point to allow for breaks. Order of assessments will be randomly determined. The proposed clinical assessments are:

1. Personal narratives measure (LUNA Discourse Protocol): proposed as possible primary outcome measure in a future trial. Participants will produce two personal narratives at each assessment point, which will be video/audio recorded. They will be given standard scripted instructions specifying the possible topics, the length and how the story should be delivered. The narrative will be spoken to the assessing therapist. S/he will not provide any cues or ask questions. S/he may request further output (‘Can you tell me more’) or check whether the story is complete (‘Have you told me everything?’). Recording will cease when the participant indicates that the story is finished. Discourse samples will be analysed according to the LUNA Research Discourse Analysis Protocol. The analysis will be conducted by non-treating therapists/researchers who are blinded to group allocation. The analysis yields the following data: number and %narrative words, and number of narrative words/minute; %CIUs; number of CIUs/minute; %complete utterances; %single versus multi-clause utterances; #1, 2, and 3 argument utterances; Predicate Argument Score; story grammar count of elements; global coherence score; local coherence score; reference chains count; and overall listener judgment. Number of narrative words is proposed as the primary discourse outcome.
2. The Western Aphasia Battery-Revised Aphasia Quotient (WAB-R AQ). This measure tests speaking, auditory comprehension, naming and repetition across 4 sections and takes 30-45 minutes to administer. It is internationally used and has been recommended as a core outcome measure for aphasia trials (Wallace et al, 2019). It provides information on type of aphasia, and severity and has been tested for remote delivery (Dekhtyar et al, 2020).
3. The Communicative Participation Item Bank (CPIB) – General Short Form [PROM]. This comprises 10 items which are rated according to the level of interference caused by the person’s condition. For example, one asks about the interference with communicating when out in the community. The measure was designed for adults with communication disorders with substantial input from individuals themselves. The short form has proven appropriate for people with aphasia (Baylor et al., 2017). We anticipate overlap in item content of the CPIB with the ALA, however, we are intentionally including this instrument as it is possible that this much shorter instrument may be more acceptable (for its brevity) and on post-hoc analysis from the interviews, may align more with participants’ perceptions of benefit from the intervention. This will contribute to decision-making for a future large trial. <15 minutes.
4. The Communication Confidence Rating Scale for Aphasia (CCRSA) [PROM]; 10 items. This is the only confidence measure in our field and is increasingly used in treatment studies. It takes <10minutes.
5. The Assessment for Living with Aphasia (ALA) [PROM]. This is a 45 item pictographic self-report test of aphasia-related quality of life, created by an internationally leading charity in Canada. It is increasingly used in treatment studies and takes up to 45 minutes.

We are removing the Warwick Edinburgh Mental Wellbeing Scale (WEMWBS) [PROM]; 14 items. We believe that the item content within will be too influenced by COVID-19 circumstances and thus will not yield useful data and will also add an unnecessary burden on participants.

***Primary and secondary outcomes***

The primary clinical outcome is discourse as measured through the LUNA Discourse Protocol. Secondary clinical outcomes include language (WAB-R AQ only); communicative participation (CPIB); communicative confidence (CCRSA); and aphasia-related quality of life (ALA).

***Profiling assessments***

*Profiling and co-variate measures:* the FAST is completed during screening; the WAB-R-AQ is used at baseline; and the Raven’s Coloured Progressive Matrices (RCPM) is used at baseline.

We have removed the Corsi block tapping assessment (Kessels et al., 2000). It is a nonverbal measure of executive working memory, however this cannot be completed remotely. We have replaced this with the RCPM which is a world-recognised non-verbal assessment of cognition. It will illustrate participants’ abilities to manage the multi-level integrated nature of the therapy used as well as the meta-linguistic and cognitive approach adopted in the intervention. It consists of 3 sets of 12 items each where the participant must match a smaller stimulus figure into a larger whole (all visual). The RCPM is embedded in the WAB-R.

**Sample size**

This is a feasibility study, so is not powered to provide definitive data about the benefits of the intervention. Our sample size is based on recommended guidance for pilot or feasibility studies i.e. 12 per group (Julious, 2005) and is also the maximum number we can achieve in the time and resources available. We are seeking to recruit 24 participants. Allowing for some attrition this will hopefully ensure that 20 complete the study.

**Data management and monitoring**

A de-identified master dataset will be created and used for all main analyses. This will be stored on a password protected server accessible only to the research team. We note that de-identified data constitutes personal data. Personal data will be stored in electronic form for 10 years after the study has ended. The dataset will be monitored and updated by the PIs at least monthly.

Audio/digital recording interviewees or video recording will be saved onto encrypted devices and only transferred via encrypted files. Direct quotes will always be de-identified using participant number but also mindful of content which might unwittingly identify participants to their regular care group.

Paper records will not be generated until after lockdown has ceased and are anticipated to be minimal. All paper-based data will be kept in a locked filing cabinet at City, University of London. Data and identifiers will be kept in separate, locked filing cabinets. All data will be stored within a secure swipe-card protected area. Paper data will be converted to electronic form as appropriate, and paper documentation shredded and disposed of in confidential waste sacks as soon as possible.

All digital files will be deidentified as far as possible (i.e. video files will not have names attached) and will be stored on password protected computer files and stored securely in OneDrive. Zoom recordings will be saved on the local device, not in cloud storage. Storage on encrypted devices (e.g. laptop, hard drive, USB) will be used for any local data transfer. To enable data sharing with the two international grant co-applicants (Boyle and Hersh) will we establish a data sharing agreement and ensure that legal safeguards are in place (e.g. standard contractual clauses). This will be led by PI Cruice with Mr Piri Norris (Information Assurance Team Leader, IT, City).

We will keep deidentified electronic data for the requisite 10 years as required by City, University of London. For linked student projects, data will be kept beyond the recommended time period (their graduation), as data is likely to contribute to an ongoing transcription reliability project, with future student research projects assessing reliability of other remaining personal narratives. We will ensure secure destruction of any electronic data uses the university's Information Services, with requests logged through IT Service Now.

**Analyses**

***Quantitative analysis***

The main quantitative analyses will be descriptive ones (n%) to ascertain feasibility parameters such as recruitment and attrition. However preliminary power calculations will be conducted bases on effect sizes (Cohens’ d and partial eta squared) to determine sample size for future full clinical trial of LUNA compared to usual care. In addition, correlational analysis will help to identify which factors associate optimum response to LUNA intervention.

***Qualitative analysis***

We will follow the recommended Standards for Reporting Qualitative Research (<https://www.equator-network.org/reporting-guidelines/srqr/>). The primary source of data is in-depth individual interviews with participants. All interviews will be recorded with consent and transcribed verbatim. We will use Framework Analysis which means raw data is tagged or coded according to a thematic index, and then data is synthesized and can be considered according to theme and case.

**Patient and Public Involvement**

LUNA has patient and public involvement embedded at its core. Four speech and language therapists and four people with post-stroke aphasia have collaborated with the research team from the outset of the grant application process. They comprise the LUNA Advisory Group meeting periodically throughout the research project and were intensively involved as co-designers in Phase 3 determining the discourse assessment and outcome measures, and the treatment. They have also contributed on treatment fidelity criteria, the model of service delivery (qualified and supported with assistant), phase 4 therapist training, and manuals. They have advised on the remote data collection methods and are working regularly with the PIs and some research staff to practise online procedures. In future meetings they will advise on issues that arise in the trial, be involved in discussion and interpretation of the findings and their dissemination, and consider ways in which to harness the emerging community of practice that is developing from this project.

**Adverse events and post-trial care**

LUNA is exploring a non-invasive, low risk intervention so adverse events arising from participation are unlikely. All members of the LUNA team (Project Manager, RAs) will report adverse events to the project PIs, regardless of whether or not these events are connected to the activities of the project. Events will be recorded on a password protected computer drive. PIs will discuss how the adverse event is being managed with the reporting staff member. For example, this might include hospitalisation or contact with Social Services. If necessary additional steps will be taken, e.g. involving referrals to relevant services.  The team may also discuss whether continued involvement in the project is in the participant’s best interest.

Participating in this study is unlikely to cause harm or discomfort to participants. We have trialled LUNA on several individuals as case studies over the past 4 years and worked extensively with 4 people with aphasia for 6 months to codesign our current LUNA programme. Telling personal stories is not considered intrusive and is indeed welcomed by our PWA team who strongly advocate working on people’s own words and language in therapy. Participants will be discouraged (if necessary) from telling their “stroke story”; although important to individuals, it is not the focus of LUNA.

The intervention is not expected to cause distress however, procedures are in place in case this occurs. Some concerns may also arise from the social isolation experienced by individuals during COVID-19 lockdown. If distress is evident the individual leading the session with the participant will pause and discuss, and either take a break or discontinue the session as appropriate. S/he will discuss the cause of the distress and will support the participant in the session. S/he will raise the situation as soon as possible with a member of the City-based research team, and jointly consider appropriate course(s) of action. S/he will discuss and agree a course of action then with the participant and implement and monitor this. This may include encouraging the participant to make contact with their GP for support, and/or contacting The Stroke Association for support (depending on the nature of the concern). Therapy assistants are not expected to be sufficiently skilled to lead this and are supervised, therefore the staff member will manage this on their behalf.

**Dissemination policy**

At the end of the study, each participant will be provided with a feedback sheet detailing his/her individual results on the outcome measures. At the end of the study, all participants will be also provided with a summary of the main findings. This will be paper-based/ written in Microsoft Word and supported with graphics and images. It will be written in a style considered accessible for people with mild to moderate aphasia. This brief brochure will be posted or emailed to participants (their preference). It will also be available publicly on the project website <https://blogs.city.ac.uk/luna>. If social isolation measures stop in time, all participants will be invited to a dissemination day at City, which will report on our main findings of the study. Our team has run several events of this kind, linked to each of our past research projects. Finally, we will write academic peer-reviewed journal articles and submit abstracts to academic conferences.

**Study status**

Recruitment will begin in June 2020 (pending full ethical approval). Data collection will continue to April 2021. No recruitment or data collection has commenced.

**References**

Agostini, M., Garzon, M., Benavides-Varela, S., De Pellegrin, S., Bencini, G., Rossi, G., Rosadoni, S., Mancuso, M., Turolla, A., Meneghello, F., & Tonin, P. (2014). Telerehabilitation in poststroke anomia. BioMed Research International, 2014, 1–6. https://doi.org/10.1155/2014/706909Telerehabilitation in poststroke anomia. *BioMed Research International*, 2014, 1–6. https://doi.org/10.1155/2014/706909

BRADY, M., KELLY, H., GODWIN, J., ENDERBY, P., and CAMPBELL, P., 2016, Speech and language therapy for aphasia following stroke. *Cochrane Database of Systematic Reviews*, **Issue 6**, Art. No: CD000425.

Cruice, M., Woolf, C., Caute, A., Monnelly, K., Wilson, S. and Marshall J. (under review)

Delivering a personalised online supported conversation for participation intervention for people with aphasia.

Dekhtyar, M., Braun, E. J., Billot, A., Foo, L., & Kiran, S. (2020). Videoconference administration of the western aphasia battery-revised: Feasibility and validity. American *Journal of Speech-Language Pathology*, 1-15. doi:10.1044/2019_AJSLP-19-00023

Enderby P, Wood V, Wade D. Frenchay aphasia screening test (FAST). West Sussex: Wiley; 1986.

Guo, Y. E., Togher, L., Power, E., Hutomo, E., Yang, Y. F., Tay, A., Yen, S.-C., & Koh, G. C.-H. (2017). Assessment of aphasia across the International Classification of Functioning, Disability and Health using an iPad-based application. *Telemedicine and e-Health*, 23(4), 312–326

Julious. 2005. Sample size of 12 per group rule of thumb for a pilot study. *Pharm Stat, 4*(4).

Kendrick et al. (2019). Executive control in frontal lesion aphasia: Does verbal load matter? *Neurospyschologica, 133*, online.

Pearl, G. & Cruice, M. (2017). Facilitating the involvement of people with aphasia in stroke research by developing communicatively accessible research resources. *Topics in Language Disorders, 37*(1), 67-84.

ROSE, M., FERGUSON, A., POWER, E., TOGHER, L., and WORRALL, L., 2014, Aphasia rehabilitation in Australia: current practices, challenges and future directions. *International Journal of Speech-Language Pathology,* **16(2)**, 169-180.

Theodoros, D., Hill, A., Russell, T., Ward, E. & Wootton, R. (2008) Assessing acquired language disorders in adults via the Internet, *Journal of Telemedicine and e- Health* 2008 552–559. pmid:18729754

WALLACE, S., WORRALL, L., ROSE, T., Le DORZE, G., CRUICE, M., ISAKSEN, J., KONG, A., SIMMONS-MACKIE, N., SCARINCI, N., and GAUVREAU, C., 2017, Which outcomes are most important to people with aphasia and their families? An international nominal group technique study framed within the ICF. *Disability and Rehabilitation,* **39(14)**, 1364-1379.

Webster et al., (2015). Is it time to stop “fishing”? A review of generalisation following aphasia intervention. *Aphasiology, 29*(11), 1240-1264.

Weidner, K., & Lowman, J. (2020). Telepractice for adult speech-language pathology services: A systematic review. *Perspectives of the ASHA Special Interest Groups*, 5(1), 326-338. doi:10.1044/2019_PERSP-19-00146

WHITWORTH, A., LEITAO, S., CARTWRIGHT, J., WEBSTER, J., HANKEY, G., ZACH, J., HOWARD, D., and WOLZ, V., 2015, NARIA: A new twist to an old tale. A pilot RCT to evaluate a multilevel approach to improving discourse in aphasia. *Aphasiology*, **29(11)**, 1345-1382.

Woolf, C., Caute, A., Haigh, Z., Galliers, J., Wilson, S., Kessie, A., Hirani, S., Hegarty, B. & Marshall, J., (2016). A comparison of remote therapy, face to face therapy and an attention control intervention for people with aphasia: a quasi-randomised controlled feasibility study. *Clinical Rehabilitation*, 30(4), pp.359-373
